# Vault changes in eyes with a vertically implanted implantable collamer lens

**DOI:** 10.1038/s41598-024-52913-8

**Published:** 2024-02-12

**Authors:** Masayuki Ouchi

**Affiliations:** Masayuki Ouchi Eye Clinic, 9-1 Nishikujo Ohkuni-cho, Minami-ku, Kyoto, 601-8449 Japan

**Keywords:** Physiology, Medical research

## Abstract

Sulcus-to-sulcus vertical diameter is longer than the horizontal. However, the effect of vertical positioning of the implantable collamer lens (ICL) on the vault compared to preoperative prediction is unclear. This prospective consecutive case series aimed to examine postoperative and preoperative predicted vaults during vertical ICL fixation. This study assessed 180 right eyes in 180 patients with myopic astigmatism. For the 90 eyes in 90 patients who underwent horizontal fixation (horizontal group) and 90 eyes in 90 patients who underwent vertical fixation (vertical group), biometrics by three-dimensional tomography of the anterior segment optical coherence tomography (A-OCT) was performed before surgery and 2 h, 1 day, 1 week, 1 month and 3 months after surgery. The anterior chamber depth did not change over time in both groups, and there was no significant difference from preoperative values. The postoperative vault values were significantly lower in the vertical group than in horizontal group over time. The difference between vault measurements and preoperative predictions at all postoperative time points was significantly greater in the vertical group than in horizontal group. Vertical fixation of the ICL reduced the vault by > 100 μm compared with horizontal fixation, which was different from the A-OCT preoperative predicted value.

## Introduction

A phakic implantable collamer lens (ICL) is widely used in refractive surgery to treat myopia and myopic astigmatism. The ICL is a rectangular lens that is centrally and axially fixed by contacting the four corners with the ciliary sulcus.

Considering the effects of a corneal incision in the center of the cornea and larger corneal diameter in the horizontal direction than in the vertical direction, it is common to insert an ICL through the temporal incision and fix it laterally. The toric model is also designed for horizontal fixation.

In addition, Biermann et al. reported that the vertical diameter is an ellipse that is on average 0.35 mm longer than the horizontal diameter in cataract patients^1^. The STAAR calculator and size determination software based on three-dimensional anterior segment optical coherence tomography (A-OCT) (CASIA2, TOMEY Corporation, Aichi, Japan), which has been widely used in recent years, is created on the premise of lateral fixation.

However, because most young patients undergoing ICL surgery have with the rule astigmatism, a vertical incision with vertical fixation is advantageous considering surgically induced astigmatism in these patients. Kamiya et al.^2^ reported safety, efficacy, and predictability for vertical fixation of an ICL using a superior incision in terms of refractive outcome and uncorrected visual acuity. Moreover, vertical fixation of the ICL may result in better rotational stability owing to its anatomical characteristics^3,4^. However, the sulcus-to-sulcus (STS) diameter may differ between the vertical and horizontal directions. The vault should be between 250 and 750 µm (µm). Some studies suggest that a vault of around 500 µm is optimal^5^.

Thus, if the size of the ICL determined based on current biometric measurements is fixed vertically as it is, the postoperative vault (corresponding to the distance between the anterior and posterior surfaces of the ICL) may differ from the preoperative predicted vault. In addition vault is an important factor affecting postoperative visual acuity and corneal endothelial cell density loss, especially in shallow anterior chamber depth (ACD) eyes^6^. Hence, ICL size selection should also be considered. Moreover, determining the characteristics of postoperative results with vertical fixation will be necessary when creating a size determination nomogram for vertical fixation. Therefore, this study aimed to examine the postoperative vault when the ICL was vertically fixed, as well as its relationship with the preoperative predicted vault, and compare vertical fixation with horizontal one.

## Results

### Basic information on implantable collamer lens insertion

Fifty-seven male individuals and 123 female individuals were enrolled in this study, with a mean age of 31.6 ± 7.4 (range, 20–45) years. All test items were acquired in all patients, and there were no missing cases.

Table 1 shows the basic preoperative data in both groups. In biometric measurements, no difference was observed between the two groups in any of the parameters of eye axial length, ACD, white-to-white (WTW) distance, corneal thickness (CT), and lens thickness. There was no difference between the two groups in uncorrected visual acuity, corrected visual acuity, or objective spherical equivalent, whereas both subjective spherical and subjective/objective astigmatism were significantly greater in the horizontal group than in the vertical group (Table 1).

**Table 1:** Preoperative baseline data.

	Horizontal group	Vertical group	P value
Age (years)	31.0 ± 6.9	32.1 ± 7.9	0.28
Male:female	29:61	28:62	0.99^※^
AL (mm)	26.75 ± 1.15	26.61 ± 1.22	0.71
ACD (mm)	3.28 ± 0.24	3.32 ± 0.25	0.54
WTW distance (mm)	11.46 ± 1.19	11.52 ± 1.02	0.66
CT (mm)	0.543 ± 0.031	0.524 ± 0.064	0.01
LT (mm)	3.81 ± 0.83	3.76 ± 0.26	0.49
UDVA (LogMAR)	1.50 ± 0.18	1.41 ± 0.35	0.07
CDVA (LogMAR)	− 0.11 ± 0.07	− 0.12 ± 0.08	0.23
SS (D)	− 8.36 ± 2.31	− 7.65 ± 2.05	0.04
AC (D)	1.08 ± 0.96	0.36 ± 0.56	< 0.001
OS (D)	− 8.39 ± 2.35	− 8.10 ± 2.03	0.08
OC (D)	1.44 ± 0.84	0.83 ± 0.79	< 0.001

Mann–Whitney U test.

*ACD* anterior chamber depth, *AL* axial length, *CDVA* corrected distance visual acuity, *CT* corneal thickness, *LT* lens thickness, *OC* objective cylinder, *OS* objective sphere, *SC* subjective cylinder, *SS* subjective sphere, *UDVA* uncorrected distance visual acuity, *WTW* white-to-white.

^※^Fisher’s exact probability test.

Regarding the ICL size distribution, 12.6 mm was the most common ICL size in both groups, accounting for > 60% of the total incretions, followed by 12.1 mm in approximately 30%, and 13.2 and 13.7 mm with no difference between the two groups. In the vertical group, non-toric models were inserted in all patients, whereas in the horizontal group, toric models were inserted in 52 of the 90 eyes (Table 2).

**Table 2:** Information of implanted ICL.

Size	Horizontal	Vertical
	Number of eyes (%)	Number of eyes (%)
12.1 mm	28 (31.1)	25 (27.8)
12.6 mm	59 (65.6)	54 (60.0)
13.2 mm	2 (2.2)	11 (12.2)
13.7 mm	1 (1.1)	0 (0)
Non-toric	38 (42.2)	90 (100)
Toric	52 (57.8)	0 (0)

*ICL* implantable collamer lens.

### Changes in postoperative biometry

**Figure 1:**
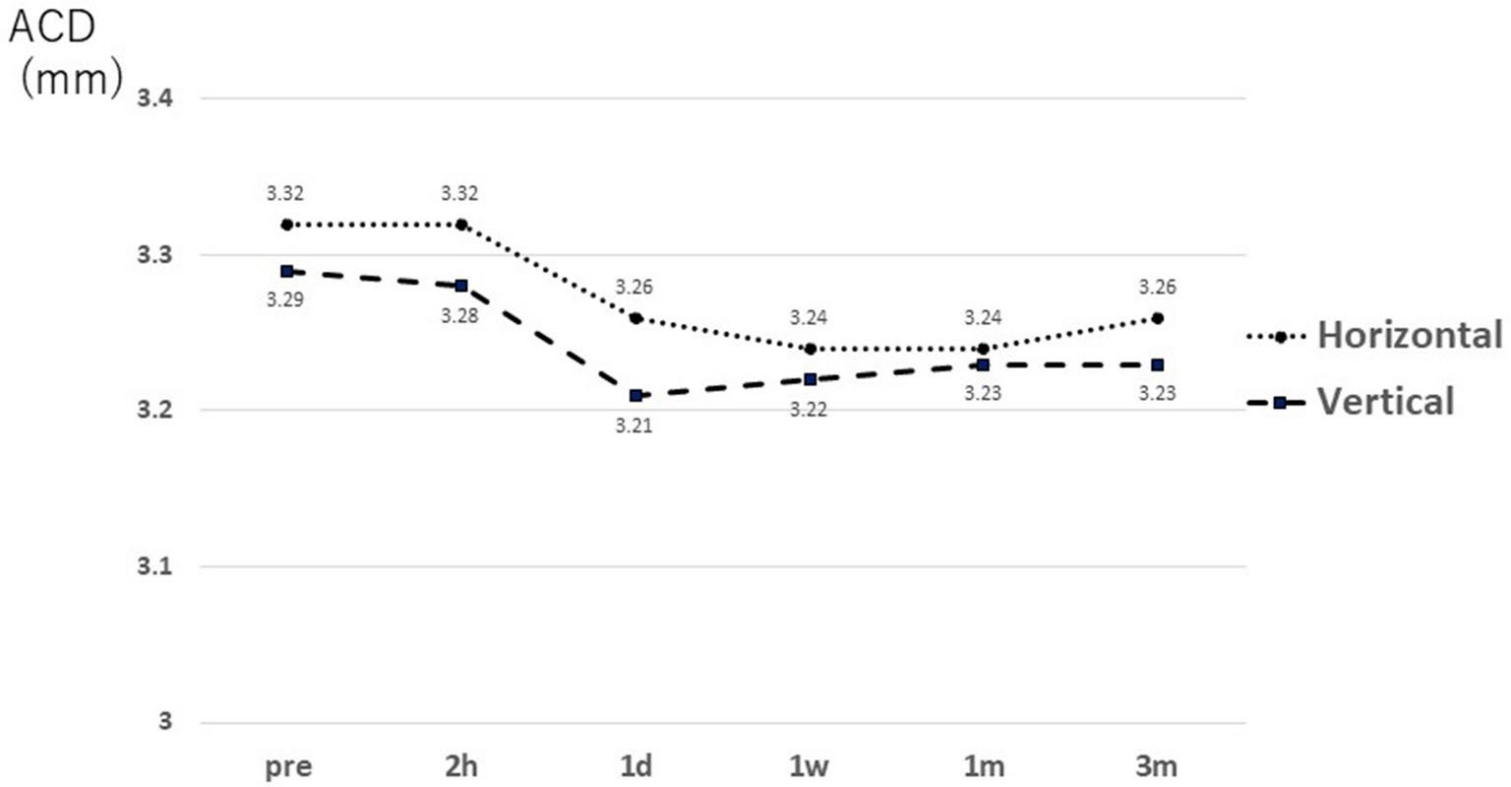
Changes in ACD over time. No change is observed in neither horizontal nor vertical fixations from before surgery to 3 month after surgery. *1d* 1 day postoperative, *1m* 1 month postoperative, *1w* 1 week postoperative, *2 h* 2 h after surgery, *3m* 3 months postoperative, *ACD* anterior chamber depth: distance from the corneal endothelium to the front surface of the lens, *Pre* before surgery.

The ACD (distance between the corneal endothelium and anterior surface of the lens) remained unchanged at 2 h (2 h), 1 day (1d), 1 week (1w), 1 month (1 m), and 3 months (3 m) in both groups, and there was no significant difference from the preoperative value at any time point (P > 0.05, Steel method) (Fig. 1).

**Figure 2:**
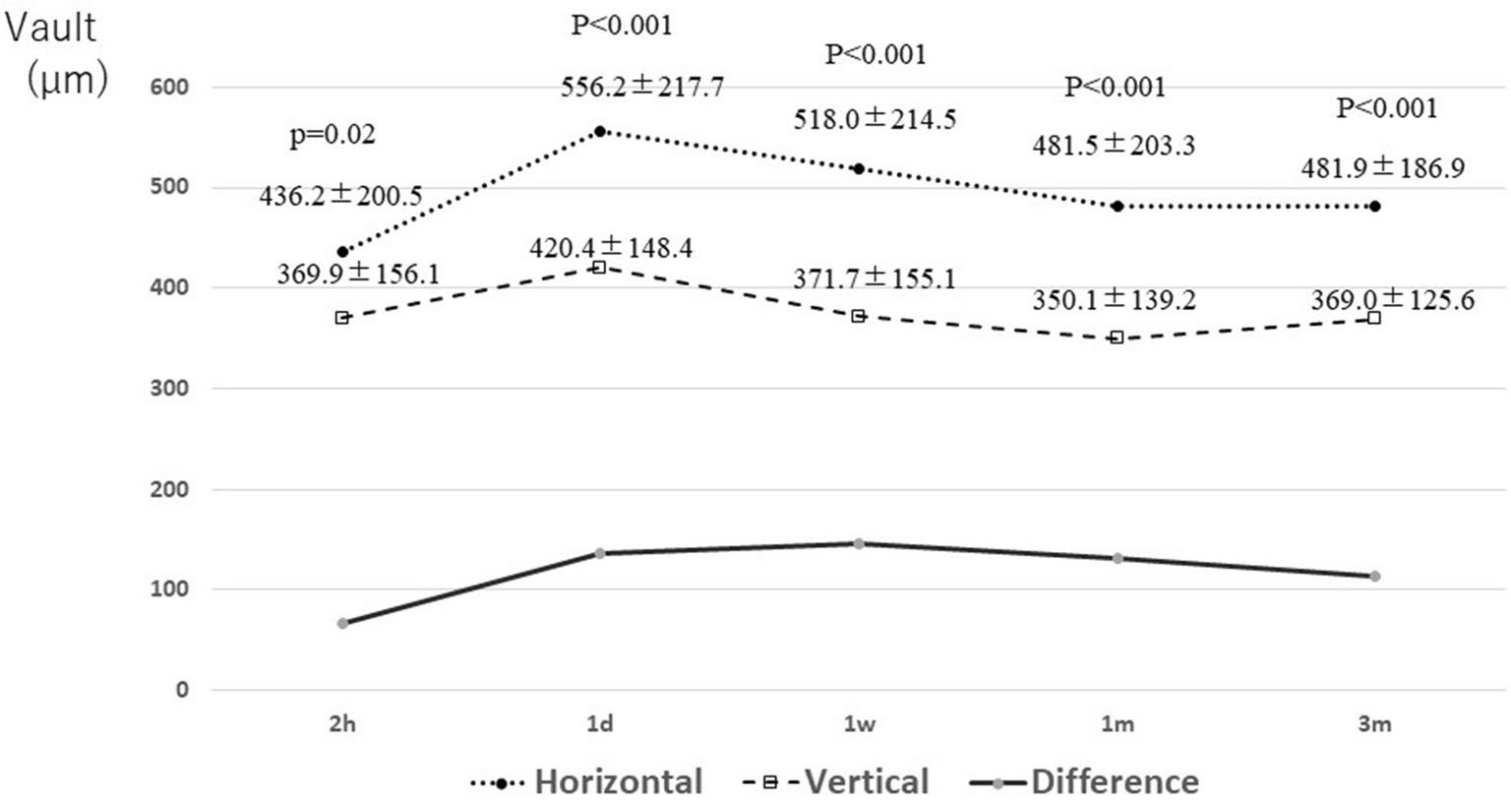
Time course of the postoperative vault. Both fixation methods exhibit similar changes over time. After 1 day, vertical fixation shows lower vault values than horizontal fixation by > 100 μm, and the difference is constant. *1d* 1 day postoperative, *1m* 1 month postoperative, *1w* 1 week postoperative, *2 h* 2 h after surgery, *3m* 3 months postoperative.

However, although both the horizontal and vertical groups exhibited similar tendencies, with the vault increasing from immediately after surgery to the next day and then gradually decreasing, the vault in the horizontal group was significantly greater throughout the course than in the vertical group, and the vault at the last observation was greater in the horizontal group (481.9 ± 186.9 µm) than in the vertical group (369.0 ± 125.6 µm) (P < 0.001). In addition, the difference between the two groups remained at approximately 110–140 µm from the next day until after 3 m (Fig. 2).

**Figure 3:**
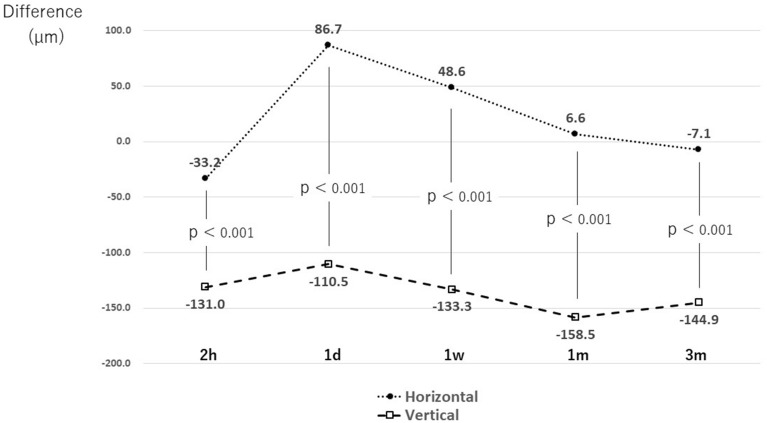
Vault difference between preoperative prediction using the KS formula and postoperative actual measurement. At the time of the final measurement, the value is close to the measured value with horizontal fixation, whereas the measured values are lower than the predicted values throughout the course with vertical fixation. *KS* Kolmogorov–Smirnov, *1d* 1 day postoperative, *1m* 1 month postoperative, *1w* 1 week postoperative, *2 h* 2 h after surgery, *3m* 3 months postoperative.

Next, considering the difference between the vault preoperative KS formula prediction value and postoperative actual measurement value for the size of the inserted ICL, in horizontal fixation, the values were slightly lower than the predicted values immediately after the operation. However, on the next day, the values were higher than the predicted values, and after 3 m, they were almost as predicted. However, for vertical fixation, the actual postoperative value was > 100 µm lower than the predicted preoperative value throughout the course (Fig. 3).

## Discussion

We investigated the ACD and vault for horizontal and vertical fixations of the ICL. There was no change in the ACD from before to 3 m after surgery in both groups. However, the vault in the horizontal fixation group was greater than that in the vertical group throughout the course, and the difference between the two groups was stable from the next day onward and was approximately 110 µm at the final measurement. Comparing the preoperative prediction using the KS formula and actual measurement after surgery, the horizontal fixation almost matched the preoperative prediction in the final measurement, whereas vertical fixation value was lower than the predicted one. The vault was low immediately after surgery, increased once on the next day, and then gradually decreased over the next month in both fixation methods.

Normally, temporal incision and horizontal fixation are standard surgical procedures used for ICL implantation.

However, because most young patients undergoing ICL surgery have with the rule astigmatism, some surgeons prefer superior incision^2^, and there are also cases where the non-toric model is fixed in the vertical orientation. Additionally, this report demonstrated safety at 1 year postoperatively^2^.

Furthermore, to take the advantage of the vertical ciliary sulcus, which is longer than the horizontal ciliary sulcus, to prevent the rotation of the toric ICL, some surgeons prepare the ICL for the reverse toric axis and fix it vertically. Kamiya et al.^2^ reported safety, efficacy, predictability, and stability of the vertical fixation of ICL in terms of refractive outcome and uncorrected visual acuity. However, in 95% of Caucasian patients, the ciliary sulcus is larger in the vertical direction than in the horizontal one^1^. Furthermore, Kojima et al. reported that postoperative vault changes following ICL surgery may also be associated with postoperative rotation^7^. This difference may affect the determination of the lens size in vertical fixation because low vault can be an incidence from anterior capsular opacification and cataract formation and excessively high vault is a risk factor of inflammation, high intraocular pressure, angle-closure glaucoma and pigment dispersion syndrome. Thus, it is important to consider the postoperative vault in the case of vertical fixation and its relationship with currently frequently used prediction formula for ICL size based on the horizontal WTW and angle-to-angle (ATA) distances. However, the abovementioned reports on vertical fixation of ICLs discuss postoperative refraction and rotational stability^2,3^ but do not mention postoperative vault.

The ICL can be manufactured in four sizes, ranging between 12.1 and 13.7 mm, and must be selected from preoperative biometric measurements. Haptics is fixed at four points on the elliptical ciliary sulcus, although it is difficult to measure the STS distance preoperatively without specialized inspection equipment, such as an ultrasound biomicroscope^4^. Moreover, the STAAR surgical calculator, which supplies ICLs, also determines the ICL size with reference to the WTW distance only. On the other hand, in cases of postoperative high vault, the technique of vertical rotation of the implanted ICL,^8^ and the option of vertical fixation of the same size lens in the second eye surgery,^9^ have also been reported. These concepts have received more attention in recent years because the central port has eliminated the need for laser iridotomies and surgical iridectomy.

However, the STS and WTW distances are correlated in emmetropic eyes, and the correlation is low in myopic eyes^1^. In such situation, CASIA2 is equipped with two types of ICL size determination software. Because the STS distance cannot be measured using this machine, the expected vault and expected ACD (corneal endothelium–ICL anterior distance) are calculated from other measurement data to determine the ICL size. The present study found that vertical fixation of the ICL reduced the vault by > 100 µm on average compared with the horizontal fixation. This corresponds to approximately 20% of the CT; thus, in cases where vertical fixation is planned from the beginning, it would be advisable to consider subtracting this value from that of the predicted postoperative vault from the current formula when determining the size. According to the manufacturer's instructions the recommended vault after ICL insertion is 250–750 μm, which is set with a considerable range. There are not many cases where the size is reduced by planning to fix it vertically, although this value is useful as a reference. However, while maintaining the aforementioned differences, changes in the vault over time for both vertical and horizontal fixations were similar to those shown in previous reports^10^, where the vault was increased from immediately after surgery to the next day and then decreased again. Therefore, there is no need to pay special attention to changes in the vault over time, depending on the direction of fixation. Factors that affect the postoperative vault include variations in the shape of the ciliary sulcus^11^, anteroposterior movement of the lens due to accommodation^12,13^, and miosis, which causes the ICL to be pushed backward by the iris, resulting in a lowered vault^14^. Regarding changes in the vault over time, Chen et al. reported that the vault was slightly high 2 h after ICL surgery, decreased significantly over the next day, and then approached its original height again over the next week and month^15^. In our study, the vault was lowered immediately after operation when the pupil was still in the state of mydriasis. Although the results were different from those reported by Kato et al.^14^ and Zhu et al.^15^, in the early postoperative period, the influence of residual viscoelastic substances appears to have been greater than the interference caused by the movement of the iris.

One of the limitations of this study is that there were unavoidable differences between patients and surgeons, especially in the aspiration and removal of viscoelastic substances in the data immediately after surgery. However, in this study, clinically useful information was obtained regarding the vault at the final observation period. Although the vault does not change significantly from 1 week to 1 month after surgery and is already stable at this time, it is also desirable to confirm vault changes throughout the postoperative period. Another fundamental problem is that the vault undergoes dynamic changes due to intraocular dynamics, including the pupillary light reflex^16–18^, so it would be ideal to measure the illuminance at the time of measurement and keep the conditions constant, but in this study, no illuminance measurement was performed. Also it should be added that this study is based entirely on Asian data.

In conclusion, when the ICL is vertically fixed, the postoperative change over time shows a similar trend to that when ICL is fixed horizontally; however, it is desirable to select the ICL size considering the vault would be lowered by > 100 µm immediately afterward and throughout the course of treatment. Ultimately, a calculation formula should be constructed for vertical fixation.

## Methods

### Ethical statements

This study was conducted in accordance with the Declaration of Helsinki, approved by the Masayuki Ouchi Eye Clinic Ethics Committee, and registered in the clinical trial registration system UMIN-CTR (UMIN000048964-20/09/2022). Written informed consent was obtained from the enrolled patients.

### Inclusion and exclusion criteria

In total, 180 right eyes in 180 patients who underwent ICL (STAAR Surgical Co., Monrovia, CA, USA) implantation using the model V4c for myopia or myopic astigmatism between January 2021 and January 2023 who were followed up for > 1 month were included in this study. The exclusion criteria were as follows: patients aged < 20 years, those aged > 46 years, and those with a subjective spherical equivalent of >  − 6.0 diopters (D).

### Patient selection

Patients were divided into the following two groups: all toric cases were assigned to a horizontal group and all non-toric cases were assigned to either a horizontal or vertical group using the sealed envelope method. Case enrollment was continued until both groups reached 90 eyes each.

### Power and size selection

The spherical and cylindrical specifications of the ICL were determined from preoperative visual acuity and subjective objective refraction using the STAAR surgical web calculator. Measurements by A-OCT were performed twice in each patient, and the size of the ICL was selected from the most recommended size in the calculation formula installed in A-OCT.

### Surgical procedures

On the day of surgery, dilating and topical anesthetic agents were applied. A model V4c or V5 ICL was implanted by one experienced surgeon (M.O.) using a 3.2-mm superior clear corneal incision after injecting a viscoelastic agent (OPEGAN 1.1, Santen Pharmaceutical Co., Ltd., Osaka, Japan) into the anterior chamber. Next, the ICL was inserted into the anterior chamber. In the vertical group, the ICL was directly inserted with no rotation, and its long axis was fixed at 90°; however, in the horizontal group, the ICL was rotated approximately 90° and fixed at 0° using a non-toric model or at 0° ± 15° using a toric model depending on each toric axis, followed by the insertion of the four corners of the ICL haptics beneath the iris.

Subsequently, the viscoelastic agent was replaced using irrigation–aspiration, followed by miotic agent administration.

### Outcome measurements

Visual acuity, subjective refraction, objective refraction, and intraocular pressure were examined before surgery and 1 day, 1 week, 1 month and 3 months after surgery. A-OCT was performed before surgery and 2 h, 1 day, 1 week, and 1 month after surgery to measure the ACD and vault. A-OCT scanning was performed in the horizontal direction, and the prediction formula described later was the initial setting calculated from the horizontal WTW and ATA distances. The ACD was detected by measuring the distance from the corneal endothelium to the anterior surface of the lens, and postoperative ACD was measured by manually tracing from the corneal endothelium to the anterior surface of the lens. The ACD, vault, and their changes over time in each group were compared between the two groups. The difference between the preoperative predicted vault in the selected size ICL and postoperative actual measurement in each eye was examined in both groups. The predicted vault before surgery was calculated using the KS formula version 4, which is a prediction formula installed in A-OCT. Each measurement result and numerical value are expressed as the mean ± standard deviation (SD).

### Sample size

A sample-size calculation was performed using an α of 0.05 and a power of 80%. A

previous study examining the postoperative vault of eyes with the ICL v4c model^13^ found a mean postoperative vault of 510.77 µm with an SD of 175.51. Considering a difference of 100 µm to be clinically significant, the study would have to include 86 eyes in both groups.

### Statistical analyses

Statistical analyses were performed using statistical software R (R Foundation, Vienna, Austria), and the normality of each data group was first confirmed using the F-test. Since none of the data groups had a normal distribution, the Mann–Whitney U test was used to compare values between the two groups, Fisher’s exact probability test was used to assess sex differences only, and the Steel method was used for multiple comparisons between preoperative and other periods. The statistical significance level was set at 5% (Supplementary Information S1).

### Supplementary Information

Supplementary Information.

## Data Availability

The datasets generated and analyzed during the current study are available from the corresponding author on reasonable request.
