# Supplementary material for: Vault changes in eyes with a vertically implanted implantable collamer lens
Source: Sci Rep. 2024 Feb 12;14:3484. doi: 10.1038/s41598-024-52913-8 (PMC10861517; doi:10.1038/s41598-024-52913-8)
Supplement: Supplementary file 1 — Supplementary Information. [file 41598_2024_52913_MOESM1_ESM.docx]

**Clinical Outcome of Vertical Fixation of Implantable Collamer Lens (ICL)**

**Project Summary:**

The study aims to evaluate the postoperative outcomes and fixation status of posterior chamber intraocular lenses (ICLs) with the vertical position. It will include patients aged 21 to 45 without ophthalmologic diseases other than refractive errors, who have undergone ICL insertion for myopia or myopic astigmatism. The study will compare the vertical fixation method with the conventional horizontal fixation in terms of postoperative vault as determined by Anterior Segment Optical Coherence Tomography (OCT):

Brand/Model: CASIA2

Manufacturer: TOMEY co. LTD

**Rationale & Background Information:**

ICLs are predominantly fixed horizontally with the sizing formula for ICL based on horizontal fixation. Given that many patients suitable for this procedure have with-the-rule astigmatism, there has been an inclination towards vertical fixation, which some believe offers superior rotational stability^1)^.

**General Information:**

Protocol ID: UMIN000048964

Application Date: 2020/09/27

Principal Investigator:

Masayuki Ouchi, Director Masayuki Ouchi Eye Clinic

9 Nishikujo Ohkuni-cho Minami-ku, Kyoto 601-8449, Japan

Contact: +81-75-6625660

**Condition:** Myopia/ Myopic Astigmatism

**Study Goals and Objective**s:

Evaluate the postoperative outcome and conditions of vertical fixation of posterior chamber phakic intraocular lens (Visian ICL). The study aims to provide insights that could contribute to the development of a sizing formula specific to vertical fixation.

**Study Design:**

Type: Interventional, non-randomized, prospective consecutive case series.

Blinding: No one is blinded.

Inclusion criteria: Age 21 to 45, Myopia/ Myopic Astigmatism cases.

Exclusion criteria: Those with ocular diseases other than refractive error and eyes with any intraoperative complication.

Primary outcomes: Postoperative refractive error, anterior chamber depth, vault comparison between actual data and estimated values using the K-S formula.

Sampling frame: Intervention and control groups, 90 eyes each.

Duration: Expected two years.

**Methodology:**

**Patient Recruitment:**

Patients visiting the clinic for refractive surgery will be provided with information about the study. Patients who express interest and meet the inclusion criteria will be further evaluated for participation.

Patient Visits Clinic

|

Provided Information About the Study

|

Expresses Interest & Meets Inclusion Criteria?

| |

No Yes

| |

　 Excluded Further Evaluation

|

Type of Lens Insertion?

/ |

Toric Non-Toric

/ |

Assigned to Horizontal Assigned using Envelope Method:

Group Automatically Horizontal or Vertical Group

|

Number of Eyes in Each Group = 90?

| |

No Yes

| |

Continue Inclusion Terminate Inclusion

**Surgical Technique**

Administer tropicamide every 15 minutes for 2 hours for dilation before surgery. During the procedure, implant either a model V4c or V5 ICL through a 3.2-mm superior clear corneal incision after introducing a viscoelastic agent (OPEGAN 1.1, Santen Pharmaceutical Co., Ltd., Osaka, Japan) into the anterior chamber. For the vertical group, directly insert the ICL without rotation, aligning its long axis at 90°. For the horizontal group, adjust the ICL around 90° and position it either at 0°for a non-toric model or between 0° ± 15° for a toric model based on its specific axis, ensuring the ICL haptics' four corners are tucked beneath the iris. Conclude the procedure by removing the viscoelastic agent with irrigation-aspiration and administering a miotic agent.

**Outcome Measurements**

Preoperative

Objective refraction test using an autorefractometer (ARK1, Nidec Corp., Gamagori, Japan).

Based on the test results, measure both uncorrected and corrected visual acuity utilizing a space-saving chart (SSC-370, Nidec Corp., Gamagori, Japan).

Determine the ICL's spherical and cylindrical specifications based on preoperative visual acuity and subjective objective refraction using the STAAR surgical web calculator.

Conduct anterior segment optical coherence tomography (A-OCT; CASIA2, TOMEY Corp., Nagoya, Japan). A-OCT measurements twice for each patient in the horizontal direction.

Select the ICL size based on the most recommended size from the STAAR calculator and the KS-formula ver.4 provided in the A-OCT system.

Postoperative

Visual acuity, subjective refraction, objective refraction, intraocular pressure are measured the day after surgery, 1 week after surgery, and 1 month after surgery.

Perform A-OCT imaging using horizontal directional scanning to measure both anterior chamber depth and vault at 2 hours after surgery, the day after surgery, 1 week after surgery, and 1 month after surgery.

Define anterior chamber depth as the distance from the corneal endothelium to the anterior surface of the lens.

For postoperative measurements, manually trace the distance from the corneal endothelium to the anterior surface of the lens.

Compare the anterior chamber depth, vault, and their temporal changes within each group.

Assess the difference between the preoperative predicted vault and the actual postoperative measurement for the selected ICL size in each eye across both groups.

**Data handling and coding for computer analysis**

Conduct statistical analysis using the R statistical software.

Begin by assessing the normality of each data group using the F test.

If both groups demonstrate normal distribution, employ the student t-test for comparison.

If groups are not normally distributed:

a. Use the Mann-Whitney U test for group comparison.

b. Apply the Fisher's exact probability test for evaluating sex differences.

c. Utilize the Steel method for multiple comparisons between preoperative and various periodic periods.

Set the statistical significance threshold at 5%.

**Compliance with Ethical Guidelines:**

This study adheres to the ethical principles outlined in the Declaration of Helsinki, ensuring the safety, well-being, and rights of the participants are of primary importance throughout the study.

Ethical Approval: The protocol for this study received full approval from the Masayuki Ouchi Eye Clinic Ethics Committee.

**Clinical Trial Registration:**

The study has been registered in the UMIN Clinical Trials Registry (UMIN-CTR).

Registration ID: UMIN000048964

Date of Registration: 27/09/2020

**Informed Consent:**

Before enrollment in the study, all participants were provided with comprehensive details about the study's objectives, procedures, potential risks, and benefits.

Written informed consent was obtained from all participants, ensuring they voluntarily agreed to participate after understanding the study's nature and potential consequences.

**Confidentiality:**

All data collected from participants will be kept confidential, with personal identifiers removed to maintain anonymity. Access to the data will be restricted to the research team, and any disclosures outside of the study will be made only with the consent of the participants or as required by law.

**Safety Reporting:**

Any adverse events or unexpected outcomes observed during the study will be promptly reported to the Masayuki Ouchi Eye Clinic Ethics Committee. Appropriate actions, including medical care and potential discontinuation of the study, will be considered based on the severity and nature of these events.

**References:**

1) Huang W, Ji Y, Zheng S, et al. The effectiveness and rotational stability of vertical implantation of the implantable collamer lens for the treatment of myopia. J Refract Surg. 38, 641–647 (2022).
